# Migraine Modulation and Debut after Percutaneous Atrial Septal Defect Closure: A Review

**DOI:** 10.3389/fneur.2017.00068

**Published:** 2017-03-20

**Authors:** Charles Stevens Leger, Joseph F. X. DeSouza

**Affiliations:** ^1^Department of Psychology, Centre for Vision Research, York University, Toronto, ON, Canada; ^2^Neuroscience Diploma, York University, Toronto, ON, Canada; ^3^Centre for Vision Research, York University, Toronto, ON, Canada; ^4^Department of Biology, York University, Toronto, ON, Canada; ^5^Canadian Action and Perception Network (CAPnet), Toronto, ON, Canada

**Keywords:** atrial septal defect closure, migraine, percutaneous closure, transcatheter closure, *de novo* migraine

## Abstract

**Introduction:**

Change in migraine headache (MH)—preexisting MH change or development of *de novo* MH—are known potential complications following percutaneous closure of atrial septal defect (ASD), but consensus on a causal trigger remains elusive.

**Objectives:**

To expose potential MH triggers linked, mainly by timing and occurrence, to the emergence of *de novo* MH or change in preexisting MH subsequent to percutaneous ASD closure (pASDC).

**Methods:**

The literature was systematically searched for studies available in English reporting MH status after pASDC published between January 1, 1990 and November 15, 2015. We determined the number and percentage of patients experiencing MH status change within 7 days post procedure and the cumulative total by final follow-up (Mdn = 12 months).

**Results:**

Twenty-five studies met the inclusion criteria, which accounted for a total of 1,646 pASDC patients. Pre-procedure MH prevalence was 8% (126/1,646). Change in preexisting MH occurred in a total of 72% (91/126), 12% (11/91) within 7-days after pASDC; within follow-up MH improved in 14% (18/126), resolved in 37% (47/126), but persisted in 63% (79/126). *De novo* MH incidence ranged between 10 (153/1,520) and 18.3% (153/836); 34% incipience (52/153) was within 7-days of pASDC; females accounted for 80% (63/79) of gender differentiated cases; of type distinguished cases, 42% (51/122) were MH without aura (MO) and 58% (71/122) were MH with aura (MA); MH improved in 10% (16/153), resolved in 24% (37/153) but persisted beyond final follow-up in 76% (116/153). Antiplatelet agents were effective modulators of MH in 44% (11/25) studies. Possible adverse MH-predisposing traits were scarce: larger ASD size reported in ~2% (39/1,646) of patients experiencing *de novo* MH or preexisting MH exacerbation; short aortic rim reported in three *de novo* MH patients; allergic response to occluder nickel alloy in four patients with MH status change from baseline (*de novo* or preexisting MH change not specified).

**Interpretation:**

Early intensification of MH status change but later amelioration (virtually paralleling stages of endothelialization), relatively high efficacy of antiplatelet agents, and the emergence of MA as the dominant *de novo* MH type favor proinflammatory triggers of MH status change after pASDC.

## Introduction

Migraine pathogenesis is a contentious, unresolved issue with rival theories implicating various neurovascular and biochemical mechanisms. Research has revealed a 3–5 mm/min propagating wave of depolarization referred to as cortical spreading depression (SD) or simply SD—neuronal excitation followed by depression—as a demonstrated substrate of migraine with aura (1–3) that is associated with initial hypoperfusion followed by hyperemia (4). SD has been linked to trigeminal system activation (3, 5–8), which is involved in pain transmission.

In a much simplified description of the trigeminal pain pathway [for an elaboration, see Noseda and Burstein (9)], trigeminal axons projecting from the meninges and associated blood vessel nociceptors (the peripheral trigeminovascular system) carry pain signals to the brainstem trigeminocervical complex comprised of the caudal trigeminal nucleus and spinal cord dorsal horns C1 and C2. Projections from this complex ascend and connect with other brainstem nuclei (e.g., the ventrolateral periaqueductal gray) before connecting with the more dorsal hypothalamic and thalamic nuclei. Since the caudal trigeminal nucleus and thalamic nuclei [with respect to the thalamus, principally, in migraine, the ventral posteromedial nucleus (VPM)] are central nervous system structures that advance processing of peripheral (trigeminovascular system) input they are referred to as the central trigeminovascular system. The VPM is a particularly important sensory relay for the face and head. The VPM thalamic nuclei project, in somatotopic fashion, to the primary and secondary somatosensory cortices (and to the insula). Efferent fibers from the VPM, for example, project to the face area of the primary somatosensory cortex (9, 10).

The trigeminal nerve ganglion and nerve endings release vasoactive neuropeptides into meningeal nociceptors; notably, substance P, serotonin, and calcitonin gene-related peptide (CGRP) (11, 12). A biochemical theory of migraine implicates vasoactive substances in migraine, such as 5-hydroxytryptamine (serotonin), and there is evidence that serotonin metabolism is impaired in migraineurs (13) and that serotonin released from aggregating platelets may be involved in the vasoconstriction and vasodilation associated with migraine headache (MH) (14). In addition, research implicates altered platelet activity in migraine (14–20).

Agreement on the SD trigger mechanism remains elusive. Genetic predisposition is a likely factor (21) but other variables are also in play. The phenomenon of SD has been induced by a variety of stimuli: introduction of tetanic electrical impulses, chemical agents, such as potassium ions (22) and glutamate, as well as hypoxia (23). Transient tissue oxidation has evoked SD in rats (5) and more recent data has intriguingly shown induced SD in mice from introduced microemboli with apparent associated hypoperfusion but without imaging (9.4T) or histological evidence of tissue infarct (17). Contrarily, dampened SD has been reported in rats through administration of CGRP-receptor antagonists (22).

Although the mechanism initiating the cascade of events involved in migraine is yet to be confirmed, migraine pain management has been advanced through intensified CGRP research. The neuropeptide CGRP is found in 50% of trigeminal neurons and in all organ systems of the body (10). There is a well-documented linkage between CGRP release and migraine (24–27), and GGRP has proven to be a factor in vasodilation of meningeal vessels (28, 29). The neuropeptide exhibits heightened levels during MH attack (29), its intravenous introduction can induce MH interictally in MA patients (30) and triptans (e.g., sumatriptan) diminish release of CGRP and are relatively effective in management of migraine pain (31). CGRP-receptor antagonists as well as monoclonal antibodies show promise for the future of MH pain management (32, 33).

For MA, here is a known association between cardiovascular risk factors such as pre-clinical brain lesions and ischemic stroke (34–39), which implicates inflammatory response as a potential trigger of SD, at least as concerns MA. Experimental hypoxia has been demonstrated to induce aura or migraine-like symptoms in migraine patients and induce migraine aura-like symptoms in healthy controls (40). In recent onset migraineurs, altered arterial measures have been documented, including reduced brachial and femoral artery compliance as well as decreased brachial artery diameter (41). Elevated vascular risk factors have been found in migraineurs, including vasoactive plasma endothelin (ET-1), a marker for atherosclerosis and endothelia injury (42). Increased carotid artery media thickness, a subclinical marker of early atherosclerosis (43, 44), has been reported as heightened in pediatric migraineurs (45) and in adult migraineurs (42).

Within the clinical context of corrective heart procedures, a procedure specific vascular-related trigger to MH surfaced when a multivariate analysis found one such type of corrective procedure was the only independent predictor of *de novo* MH onset (46). In brief, the corrective procedure in general involves non-surgical repair of an atrial septal defect (ASD). An ASD is a deficit in the interatrial septum resulting in a gap or hole between the right and left atria enabling abnormal interatrial blood flow and mixing of right atrial venous and left atrial arterial blood. The corrective procedure is referred to as percutaneous (or transcatheter) ASD closure (pASDC) and involves imaging guidance [typically transesophageal echocardiography (TEE)] of a catheter inserted in the femoral vein and directed to the interatrial septum. The catheter has an attached occluder device that is maneuvered to plug the interatrial hole. Once the occluder is deemed securely and correctly positioned, the catheter is withdrawn (47). Patients are typically started on antithrombotic 24 h before the procedure and continue antithrombotic therapy for 3–6 months after the procedure. The percutaneous ASD closure (pASDC) method of treatment has a track record of positive cardiac remodeling and high safety and efficacy (48–51) as well as high rates of closure success including a long-term closure rate verification of 97% (52). ASD prevalence is 1–3 in 10,000 and is more common in females than males, with a female to male ASD ratio of 1.6 (53). Migraine prevalence is also higher in women, about three times higher, with the peak incidence between 25 and 55 years of age (54).

Change in preexisting MH (exacerbation, improvement, complete resolution) or development of *de novo* MH, are broadly acknowledged neurological alterations that can follow pASDC. However, the prevalence in patient frequency of MH change within days to months after pASDC has not been formally reviewed across studies.

This review focuses on migraine, its *de novo* appearance or modulation, in the context of the corrective coronary procedure pASDC. The objective was to shed further light on potential MH triggers by examining qualitative data (patient frequency and percentage) from the literature to assess the influence of pASDC and other pertinent clinical (e.g., effectiveness of antiplatelet agents) and demographic data on change in MH status. Of particular interest was the proportion of patients who experienced *de novo* MH onset within 7 days from pASDC. New onset of MH occurrence constrained within this short time interval has minimized likelihood of procedure-extraneous confounds and provides a unique window to the pathogenesis of migraine, at least as it occurs within this context. Procedure-related events (e.g., proinflammatory response factors) loom large as potential MH triggers. In addition, the proportion of patients in whom MH resolved within follow-up (median of 12 months) or persisted after follow-up was also determined as these are variables that further characterize migraine and its potential underlying mechanisms. Such time-to-event measures provide an indication of the extent to which stages of healing, notably endothelialization, parallel and potentially contribute to MH status change. Additional differentiation of MH status change after pASDC by gender and type was also extracted from the data, but it was restricted in the literature, and consequently in this review, to subsets of *de novo* MH patients. Finally, an overview was included of biochemical, microembolic, and allergy factors ostensibly informing on MH.

## Methods

### Literature Search

There is no prior protocol for the current review. Cochrane databases, Google Scholar, PubMed, and Web of Science were systematically searched for relevant primary clinical observation and research data published between January 1990 and December 2015. The search arguments used were ((atrial septal defect) in Title)) *and* ((migraine) in Title), ((percutaneous) in Title) *and* ((atrial septal defect) in Title) *and* ((migraine) in Title), ((transcatheter) in Title) *and* ((atrial septal defect) in Title) *and* ((migraine) in Title), ((atrial septal defect) in Title) *and* ((closure) in Title), ((atrial septal defects) in Title) *and* ((migraine) in Title), ((atrial septal defects) in Title) *and* ((headache) in Title), ((atrial septal defect) in Title) *and* ((headache) in Title), ((interatrial shunts) in Title) *and* ((migraine) in Title), ((percutaneous) in Title) *and* ((atrial septal defect) in Title) *and* ((migraine) in any field, ((atrial septal defects) in Title) *and* ((transcatheter) in Title) *and* ((migraine) in any field). For background information, the literature was also searched for ((patent foramen ovale) in Title) *and* ((migraine) in Title) as well as for migraine theories. To provide background on migraine in general, migraine not associated with pASDC, additional data were retrieved from Google Scholar, PubMed, and Web of Science for migraine pathogenesis and management in general with search arguments including ((migraine) in Title) *and* ((pathogenesis) in Title) as well as ((migraine) in Title) *and* ((management) in Title).

### Study Selection and Inclusion Criteria

This review sought evidence linking pASDC and migraine status change: change after pASDC in status of preexisting MH as well as the occurrence of *de novo* MH inception. Preexisting MH status change was defined mainly as change in frequency or severity or MH type. Study screening and inclusion criteria were as follows: (1) assessment of potential migraine status change—alteration of prior MH or *de novo* MH—after pASDC; (2) diagnosis of migraine by qualified personnel (i.e., neurologist, cardiologist), and preferably employing International Headache Society (IHS) migraine criteria (55, 56); (3) an English language version of the report; (4) data sufficiently explicit and complete for qualitative data review. With respect to point (2), absence of IHS criteria did not exclude a report from inclusion. As regards point (4), this was a qualitative review simply because there was insufficient appropriate quantitative data across studies for analysis of MH status change after pASDC.

### Measures

The number (patient count) and percentage of patients experiencing MH status change after pASDC was calculated for preexisting and *de novo* MH patient categories, but more data were available for *de novo* MH. Specifically, data differentiating MH status change by gender and type after pASDC was only available for *de novo* MH. Change in preexisting MH status after pASDC differentiated by gender or type was too scarcely reported to include as a review-wide representative measure. The categories of qualitative data available for change in preexisting MH were pre-procedure MH type (MO and MA), change in preexisting MH within 7 days after pASDC, change in preexisting MH by final follow-up months later, and improvement or resolution in preexisting MH by final follow-up. With the exception of pre-procedure MH type, *de novo* MH data categories were the same but *de novo* MH was additionally differentiated by MH type and gender, though such differentiation was not consistent across all studies. *De novo* MH incipience within 7 days from pASDC differentiated by type was also recorded. All categories are listed in Table 1. The effect of pASDC on MH change was also informed by individual study characterizations of MH change. Additional data items extracted where possible were mean age, mean follow-up (months), gender ratio (female to male), antiplatelet agents, rate of successful ASD closure, occluder device type used, and use of IHS migraine diagnostic criteria (see Table 3).

**Table 1:** **(A): prevalence of PEMH change (preexisting MH change: change in frequency, severity or type of migraine) after pASDC in patients with a history of MH; (B): prevalence *de novo* MH after pASDC**.

	Number of studies	Percentage fractions	*n*/*n*	%
**(A) Preexisting MH category**				
PEMH Hx	12	PEMH Hx/Grand N	126/1,646	8
PEMH MO	7	PEMH MO/PEMH Hx	55/126	44
PEMH MA	9	PEMH MA/PEMH Hx	46/126	37
PEMH CHG	12	PEMH CHG/PEMH Hx	91/126	72
PEMH CHG Wi 7d from pASDC^a^	6	PEMH CHG Wi 7d/PEMH CHG	11/91	12
PEMH Impr Wi FUP	6	PEMH Impr Wi FUP/PEMH Hx	18/126	14
PEMH Res Wi FUP	9	PEMH Res Wi FU/PEMH Hx	47/126	37
**(B) *De novo* MH category**				
*De novo* MH	18	*De novo* MH/Grand N	153/1,646	9
*De novo* MO^b^	8	*De novo* MO/*De novo* MH	51/122	42
*De novo* MA^b^	12	*De novo* MA/*De novo* MH	71/122	58
*De novo* MH Wi 7d from pASDC	11	*De novo* MH Wi 7d/*De novo* MH	52/153	34
*De novo* MO Wi 7d from pASDC	3	*De novo* MO Wi 7d/*De novo* MH Wi 7d	6/52	12
*De novo* MA Wi 7d from pASDC	8	*De novo* MA Wi 7d/*De novo* MH 7d	25/52	48
*De novo* MH F^c^	11	*De novo* MH F/*De novo* MH	63/79	80
*De novo* MH M^c^	7	*De novo* MH M/*De novo* MH	16/79	20
*De novo* MH Impr Wi FUP	2	*De novo* MH Impr Wi FUP/	16/153	10
		*De novo* MH		
*De novo* MH Res Wi FUP	10	*De novo* MH Res Wi FUP/	37/153	24
		*De novo* MH		

*^a^There were 12 studies indicating PEMH CHG, but only 6 specified migraine headache (MH) change within 7 days from percutaneous ASD closure (pASDC)*.

*^b^Type of migraine was not differentiated in 31 patients across 7 studies*.

*^c^In 74/153 patients (48%), gender of those presenting with *de novo* MH was not specified*.

*De novo MH, new-onset migraine; Grand N, total across patient number (1,646); F, female; M, male; MA, migraine with aura; Impr, improved; MH, migraine headache; MH Hx, migraine history; Impr, improvement; MO, migraine without aura; n, patient count; Nbr, number; Pat, patient; pASDC, percutaneous atrial septal defect closure; PEMH, preexisting MH; Res, resolved; Wi, within; 7d, 7 days*.

### Qualitative Data Analysis

Qualitative findings were expressed in frequencies (number of patients falling into the aforementioned categories) and percentages. Values represent the number of patients not the number of episodes. Data were tabulated and graphed where appropriate. A frequency distribution of the variables of primary interest noted in the previous section was also calculated (Table S1 in Supplementary Material). Data analysis was completed in R version 3.2.4.

## Results

A total of 60 studies were returned by a search of Cochrane databases, Google Scholar and PubMed. After removing duplicates, there were 34 articles. Of the 34 articles, one study was not included because it could not be accessed (86) at the time of this writing. Two of the articles were not included because they did not report on MH in the context of ASD (74, 87); these two articles were used for additional background information. Three additional articles were not included because, although relevant, at the time of this review they were not available in English (88, 89). Two of the articles were commentaries (90, 91) and discarded. There were 25 articles remaining that adequately fulfilled the inclusion criteria.

### Prevalence of Migraine after Percutaneous ASD Closure

Twenty-five articles based on original research represented the prevalence of migraine and accounted for 1,646 patients who underwent pASDC. After pASDC, patients experiencing change in preexisting MH was reported in 12 of 25 studies and those experiencing *de novo* MH was reported in 18 of 25 studies; 7 studies reported patients with both change in preexisting MH and *de novo* MH (19, 52, 61, 64, 71, 76, 81). In terms of study type, change in preexisting MH was reported in 3 of 8 case studies (62, 70, 82); 2 of 3 prospective studies (71, 84), and in 9 of 13 retrospective studies (19, 52, 59–61, 63, 64, 76, 81). *De novo* MH was reported in 5 of 8 case studies (57, 73, 75, 78, 92), in 2 of 3 prospective studies (71, 72), and in 11 of 13 retrospective studies (19, 46, 51, 52, 58, 61, 64, 67, 77, 81, 93). Finally, a recent randomized controlled trial reported *de novo* MH following pASDC (79).

**Figure 1:**
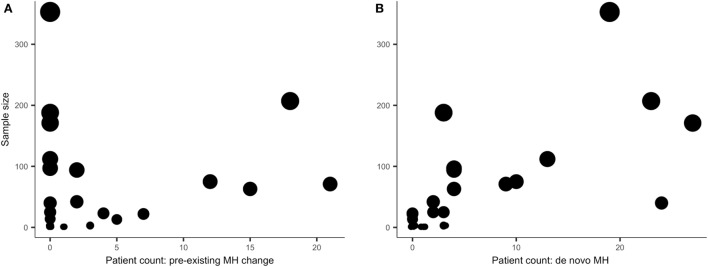
**The *y*-axes reflect study sample size; the *x*-axes show patient count (the number of patients) of those who experienced migraine headache (MH) status change after pASDC**. Some studies reported similar incidence of MH status change and to limit overplotting of duplicate patient counts the data were jittered.

Overall, *de novo* MH data were more frequently (and explicitly) reported than change in preexisting MH data. This is conveyed by a visual inspection of Figure 1. The circles in Figure 1 are scaled to reflect individual study sample size (*n*) on the *y*-axes and the *x*-axes indicate patient count. The left graph (Figure 1A) reflects the sample size and number of patients in whom change in preexisting MH differed (in frequency or severity) from baseline MH and the right graph (Figure 1B) reflects the sample size and number of patients in whom *de novo* MH occurred. Clearly, there are more studies reporting zero change in patients (on the *x*-axis) with preexisting MH in Figure 1 relative to studies reporting zero *de novo* MH illustrating that there was more *de novo* MH data available for this review. The circles in Figure 1 also convey a measure of distribution consistency among studies reporting change in MH status, as larger studies tend to show higher counts of change in preexisting MH or *de novo* MH.

Where provided by the literature, relevant continuous data with significant results are highlighted in this review on a study-by-study basis (see Results subsection Study Highlights). A summary of findings for change in preexisting MH will be reviewed first followed by a summary of findings for *de novo* MH occurrence (see Frequencies and Percentages). This will be followed by précises of relevant individual study highlights arranged by category (preexisting MH or *de novo* MH) and study-type: case, prospective, retrospective, and a single random controlled trial (RCT).

### Frequencies and Percentages

Table 1 summarizes overall MH prevalence; frequencies and percentages are based on the number of patients in a given category. Table 2 provides individual study details of the patient count and percentage in categories arguably most demonstrative of potential change in MH status after pASDC. Frequency distributions are provided in Table S1 in Supplementary Material. Note, to reduce the size of Table 2, six after pASDC measures were omitted: the breakdown of *de novo* MH patient number by gender, number of patients with prior MH who experienced improvement, the number of *de novo* MH patients who experienced improvement, the number of *de novo* MO, and MA patients who experienced *de novo* MH within 7 days from pASDC. However, these data can be derived from Table S1 in Supplementary Material. Also in aid of reducing table size, the breakdown of pre-procedure MH by type in those with preexisting MH was omitted in Table 2 and Table S1 in Supplementary Material, but these data are available upon request.

**Table 2:** **Migraine prevalence: individual study details**.

Reference	Study type	Study *n*	PEMH MH Hx: *n* (%) of study *n*	PEMH MH CHG: *n* (%) of PEMH MH	PEMH MH CHG Wi 7d. pASDC: *n* (%) of Per MH CHG	PEMH MH resolved Wi FUP: *n* (%) of PEMH MH	*De novo* MH: *n* (%) of study *n*	*De novo* MO: *n* (%) of *de novo* MH	*De novo* MA: *n* (%) of *de novo* MH	*De novo* MH Wi 7d pASDC: *n* (%) of *de novo* MH	*De novo* MH resolved Wi FUP: *n* (%) of *de novo* MH
Yankovsky and Kuritzky (13)	Case	1	1 (100)	1 (100)	1 (100)	1 (100)	0	0	0	–	–
Rodes-Cabau et al. (57)	Case	1	0	0	–	–	1 (100)	0	1 (100)	1 (100)	1 (100)
*Yew and Wilson* (58)	Retro	25	–	–	–	–	2 (8.0)	–	–	1(50.0)	1 (50.0)
Wilmshurst et al. (19)	Retro	71	21 (29.6)	21 (100)	–	–	9 (12.7)	7 (77.8)	2 (22.2)	–	–
Azarbal et al. (59)	Retro	23	7 (30.4)	4 (57.1)	–	3 (42.9)	0	0	0	–	–
Kedhi and Vermeersch (60)	Retro	14	–	–	–	–	0	–	–	–	–
Mortelmans et al. (61)	Retro	75	22 (29.3)	12 (54.5)	–	12 (54.5)	10 (13.3)	3 (30)	7 (70.0)	–	6^c^ (60.0)
Riederer et al. (62)	Case	1	1 (100)	1 (100)	1 (100)	3	0	0	0	–	–
Sharifi et al. (63)	Retro	13	5 (38.5)	5 (100)	5 (100)	5 (100)	0	0	0	–	–
Wertman et al. (64)^a^	Retro	42	2 (4.8)	2 (100)	–	–	2 (4.8)	–	–	–	–
*Fernández-Mayoralas et al*. (65)	Case	97	0	0	–	–	4 (4.1)	0	4 (100)	4 (100)	4 (100)
*Li* (66)	Retro	188	0	0	–	–	3 (1.6)	–	NA	2 (66.7)	3 (100)
Providencia et al. (67, 68)	Retro	25	0	0	–	–	3 (12.0)	–	NA	–	2 (66.7)
*Rodés-Cabau* (46, 69)	Retro	112	0	0	–	–	13 (11.6)	4 (30.8)	9 (69.2)	6 (46.2)	4 (30.8)
Castellini et al. (70)	Case	3	3 (100)	3 (100)	1 (33.3)	2 (66.7)	0	0	0	–	–
Luermans et al. (71)	Pros	63	23 (36.5)	15 (60.9)	–	13^e^ (56.5)	4 (6.3)	2 (50.0)	2 (50.0)	4 (100)	–
*Knepp et al*. (52)	Retro	94	2 (2.1)	2 (100)	–	2 (100)	4 (4.3)	–	NA	–	–
Riederer et al. (62)	Pros	22	10 (59.1)	7 (70.0)	1 (14.3)	3 (30.0)	0	0	0	0	–
Wei et al. (72)	Pros	40	0	–	–	–	24 (60.0)	11 (45.8)	13 (54.2)	–	7 (29.2)
Benemei et al. (73)	Case	1	0	0	–	–	1 (100)	0	1 (100)	1 (100)	0
*Vijarnsorn et al*. (51, 74)	Retro	353	–	–	–	–	19 (5.4)	–	NA	–	–
Kato et al. (75)	Case	1	0	0	–	–	1 (100)	0	1 (100)	1 (100)	1^d^ (100)
*Kato et al*. (76, 77)	Retro	207	29 (14.0)	18 (62.1)	2 (11.1)	6 (20.7)	23 (11.1)	10 (43.5)	13 (56.5)	13 (56.5)	8 (34.8)
*Armstrong et al*. (78)	Case	3^b^	0	0	–	–	3 (100)	1 (33.3)	2 (66.7)	3 (100)	0
Rodés-Cabau (79, 80)	Random controlled trial	171	0	0	–	–	27 (15.8)	13 (48.1)	14 (51.9)	16 (59.3)	–
	Totals	1,646	126	91	11	47	153	51	71	52	37

*The first author is italicized in studies including pediatric patients*.

*The n (%) values are individual study patient number and percentages within the specified categories. Percentages reflect a given category’s proportion of patients from that study’s total number of patients*.

*^a^After closure, five patients were reported with either exacerbated MH or de novo MH, but the breakdown was not specified. To reduce bias, an equal number of two patients were allocated to each category (PEMH MH CHG, which includes exacerbation and improvement, and de novo MH)*.

*^b^While there was a total of four patients, three patients had percutaneous ASD closure and one underwent surgical closure*.

*^c^Later follow-up was provided by another study from the same camp (81) monitoring the same patients*.

*^d^While MH resolved aura persisted*.

*^e^This percentage is likely imprecise as MH status of ~5 of the original 23 patients was unknown at final follow-up*.

*The dash (–), data not available or not applicable; CHG, change; De novo MH, new-onset migraine; FUP, follow-up; Hx, history; MA, migraine with aura; MH, migraine headache; MH, migraine headache; MO, migraine without aura; pASDC, percutaneous atrial septal defect closure; PEMH, preexiting MH; Pros, prospective; Retro, retrospective; Wi, within; 7d, 7-days*.

**Figure 2:**
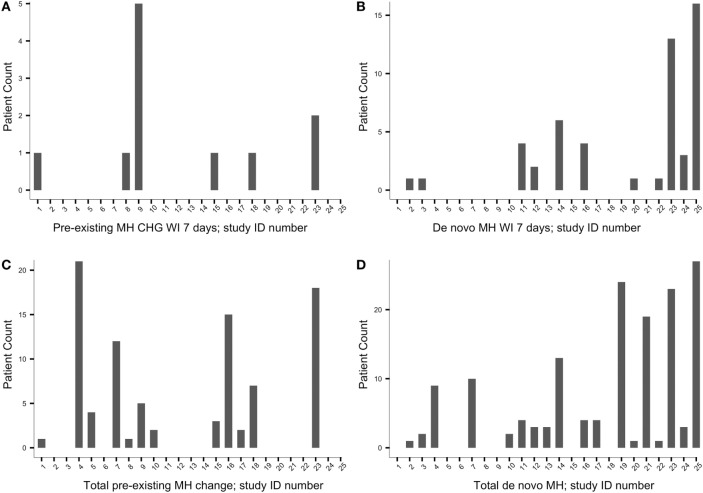
**The *y*-axes reflect patient count of migraine headache (MH) status change after pASDC; the numbers in the *x*-axes represent the study that corresponds to a given bar, and the studies that correspond to the *x*-axes numbers are listed sequentially in Table 2**. Left side graphs: **(A)** preexisting MH change patient count within 7 days from closure; **(C)** cumulative preexisting MH change patient count by final follow-up; Right side graphs: **(B)** *de novo* MH patient count within 7 days from closure; **(D)** cumulative *de novo* MH by final follow-up. CHG, change.

Recall, as specified in Section “Methods” (see Measures), two time points were set from which patient count of change in MH status was derived: the frequency of patients experiencing change in MH status within 7 days from pASDC and the total cumulative number at final follow-up. Patient count of MH status change at these two time points is represented in Figure 2. Figure 2 *y*-axes reflect patient count of MH status change after pASDC; the numbers in Figure 2 *x*-axes represent the study that corresponds to a given bar; corresponding studies are listed sequentially in Table 2. In the left side graphs of Figure 2, panel (A) depicts patient count of MH change within 7 days from closure in patients with preexisting MH; panel (C) depicts the patient count of MH change cumulatively by final follow-up in those with preexisting MH. On the right side of Figure 2, panel (B) depicts patient count of *de novo* MH within 7 days from closure, and panel (D) depicts the cumulative patient count of *de novo* MH by final follow-up.

Of the 1,646 patients, 126/1,646 (8%) had a history of MH prior to pASDC. There were 55/126 patients with MO (44%) and 46/126 patients (37%) with MA. Type of MH was not differentiated in 25/126 patients (20%) with a prior history of MH. Change in preexisting MH occurred in 91/126 patients (72%) with a prior history of MH after pASDC. These 91 patients constituted the preexisting MH change group in whom change in MH status occurred within follow-up. As noted in Section “Methods,” breakdown of post pASDC change in preexisting MH either by gender or MH type was not available across studies. For patients with a prior history of MH, change in preexisting MH occurred in 11/91 patients (12%) within 7 days after pASDC and the remaining 80 patients experienced MH change within follow-up. Preexisting MH improved in 18/126 (14%) within follow-up and resolved in 47/126 patients (37%) within follow-up.

*De novo* MH occurred in 153/1,646 patients (9%). The proportion of *de novo* MH relative to those with no prior history of migraine ranged between two percentages. While 836 patients were specified as without any prior MH history, there were also 684 patients in whom MH history status was not explicitly specified. The proportion of *de novo* MH in those with no prior MH history lies between a lower estimate of 153/1,520 (10%) and a higher estimate of 153/836 (18%). Within 7 days of pASDC, *de novo* MH onset occurred in 52/153 patients (34%), and the remaining 101/153 patients (66%) with *de novo* MH accrued within follow-up. The type of *de novo* MH was not distinguished in seven studies, which collectively accounted for 31/153 patients (20%). In the 122 cases where MH type was differentiated, *de novo* MO incipience occurred in 51/122 patients (42%) and *de novo* MA incipience occurred in 71/122 patients (58%). *De novo* MO within 7 days from pASDC accounted for MH debut in 6/52 patients (12%) and *de novo* MA within 7 days from pASDC accounted for MH debut in 25/52 patients (48%). *De novo* improvement but not complete resolution occurred in 16/153 patients (10%) and MH resolution occurred in 37/153 *de novo* MH patients (24%) within follow-up.

In those patients with a prior history of MH in whom MH persisted at final follow-up, data indicated significant reductions in episode frequency or severity (59, 61, 71, 81) or a course of gradual decreased frequency and overall improvement (19, 62, 76, 84). Nevertheless, preexisting MH persisted beyond final follow-up in 79/126 (63%) of patients with a prior history of MH. Similarly, in *de novo* MH patients, indications were for significant reduction (81) or a course of gradual decreased frequency and general improvement (46, 72, 76, 81), but MH persisted beyond final follow-up in 116/153 (76%) patients. Instances of *de novo* MH perpetuating beyond 2 years [(78): three pediatric patients], 5 years [(58): one pediatric patient], and 7 years [(73): one adult] were reported.

Eight of 25 studies included pediatric patients (46, 51, 52, 58, 76, 78, 92, 93). Occurrence of *de novo* MH has been reported to not differ significantly between pediatric and adult patients (51) (Vijarnsorn Beneficial 2012), though, antithetically, other research reports higher *de novo* MH prevalence among pediatric patients (46, 76) and younger adult patients (46, 61). Some data indicated adults who underwent change in preexisting MH were younger that those in whom MH did not persist or develop (61).

As concerns other data not tabulated in this review but potentially germane to MH status, ASD clinical characteristics [pulmonary-to-systemic flow ratio (Qp/Qs), ASD size, occluder size, etc.] generally did not differ significantly between those who did and those who did not experience pASDC migraine status change—change in preexisting MH or *de novo* MH. However, Wei et al. (72) reported significantly larger ASD size and ASD length to occluder length ratio in *de novo* MH patients, and Mortelmans et al. (61) reported significantly larger ASD size in *de novo* patients (and in patients with preexisting MH change after pASDC). There was a 93% rate of successful ASD closure within follow-up, and major adverse events were rare. Adverse events were defined as evidence within follow-up of device thrombus formation, embolism, requirement of cardiac surgical intervention (perhaps due to occluder migration), pericardial effusion, stroke, or death. Evidence of device thrombus formation was reported in just two studies (52, 93), involved two patients, one from each study: within 10 days, one patient’s thrombus disappeared with intensified administration of anticoagulant; the other patient was a 25-month-old infant with a large ASD and a respiratory comorbidity, and this patient expired. In three patients, device migration occurred requiring surgical explantation (93).

Individual study demographic and clinical characteristics are shown in Table 3 and include mean age (which varied considerably given inclusion of adult and pediatric patients), mean follow-up time (months); female to male ratio, antithrombotics, percentage of Amplatzer occluder devices used, percentage of successful pASDC, and the MH diagnostic reference used. Mean follow-up time ranged from 3 to 73 months (Mdn = 12). The female to male ratio of pASDC patients was 1,263/533 (2.37). Unfortunately, change in preexisting MH differentiated by gender was too inconsistently documented to determine proportionate MH change after pASDC by gender in those with a prior history of MH. With respect to *de novo* MH patients, new-onset MH was differentiated by gender in 79/153 *de novo* patients (52%): females accounted for 63 and males 16 of the *de novo* MH patients, which means a percentage difference of 80% (63/79) *de novo* MH occurred in females and 20% (16/79) in males. In 74/153 patients (48%) with *de novo* MH, gender of patients with MH debut was not specified. Effective modulation of MH status by antithrombotics was expressly reported in 11/25 or 44% of studies (19, 57, 63, 64, 70, 72, 73, 76, 78, 79, 82). Acetylsalicylic acid (ASA) was used in 21/25 (84%) studies, and the thienopyridine clopidogrel was used in conjunction with ASA in 12/25 (48%) of studies. The IHS criteria for migraine assessment was adopted in 17/25 (68%) studies and the Amplatzer™ septal occluder was used in 95.5% of patients; note, in the highlights that follow, use of IHS criteria and Amplatzer™ occluder devices in a given study should be assumed unless otherwise stipulated. Further, and as concerns the highlights that follow, demographic and clinical characteristics from Table 3 are not reiterated unless, according to a given study, they lent purported weight to the main outcome measure of migraine status change after pASDC.

**Table 3:** **Demographic and clinical characteristics**.

Reference	Study *n*	Mean age (SD)	Mean FUP (mos)	Female:male ratio	Anti-thrombotic	Successful ASD closure Wi FUP %	Amplatzer ASO%	MH diagnostic reference
Yankovsky and Kuritzky (82)	1	48	6	0:1	Acetylsalicylic acid (ASA); PPL	100.0	100.0	–
Rodés-Cabau (57)	1	32	12	0:1	AMITRIP	100.0	100.0	International Headache Society (IHS)
*Yew* (83)	25	16	58	18:7	ASA	100.0	100.0	–
Wilmshurst et al. (19)	71	†	6	31:40	ASA, CLOP	–	80.0	IHS
Azarbal et al. (59)	23	41 (15)	12	16:7	ASA, CLOP	–	100.0	MIDAS
Kedhi and Vermeersch (60)	14	–	NA	NA	–	–	–	–
Mortelmans et al. (61)	75	51 (19)	29	59:16	–	–	100.0	IHS
Riederer et al. (62)	1	27	3	1:0	ASA	100.0	100.0	IHS
Sharifi et al. (63)	13	32 (9)	9	9:4	ASA, CLOP	–	100.0	IHS
Wertman et al. (64)	42	–	–	NA	ASA, CLOP	–	100.0	MIDAS
*Fernández-Mayoralas et al*. (65)	97	11.75 (5.31)	9	1:3	ASA, IBP	100.0	100.0	IHS
*Li* (66)	188	24.8 (17.9)	6	136:55	ASA	96.9	100.0	–
Providencia et al. (67, 68)	25	37.8 (17.7)	12	11:14	–	–	100.0	IHS
*Rodés-Cabau et al*. (46, 69)	112	39 (19)	24	110:75	ASA, CLOP	100.0	100.0	IHS, MIDAS
Castellini et al. (70)	3	30.67 (11)	33	2:1	ASA, FNZ, LTG	100.0	100.0	IHS
Luermans et al. (71)	63	47.3 (16.4)	9	53:15	ASA, CLOP	96.3	91.2	IHS
*Knepp et al*. (52)	94	^a^	73	–	ASA	97.0	100.0	–
Riederer et al. (84, 85)	22	50 (16)	12	16:9	ASA, CLOP, PPC	100.0	100.0	IHS
Wei et al. (72)	40	25.8 (2.4)	3	33:7	ASA, CLOP	90.0	100.0	IHS
Benemei et al. (73)	1	28	24	1:0	ASA, CLOP, TICLID	100.0	100.0	IHS
*Vijarnsorn et al*. (51, 74)	353	36^b^ (13.23)	12	^c^	ASA	98.8	100.0	–
Kato (75)	1	35	24	1:0	ASA, CLOP	100.0	100.0	IHS
*Kato* (76, 77)	207	27	41	146:61	ASA, CLOP TICLID	100.0	100.0	IHS
*Armstronget al*. (78)	3	8.5 (5.23)	24	2:1	ASA	–	100.0	IHS
Rodés-Cabau (79, 80)	171	49 (15)	3	106:65	ASA, CLOP	99.4	100.0	IHS, MIDAS

*The first author is italicized in studies including pediatric patients*.

*^a^Two age groups, pediatric (n = 52): M = 7.5, SD = 4.6; adults (n = 42): M = 49.9, SD = 15.4*.

*^b^There were three age groups: children < 18, adults 18–50, adults > 50*.

*^c^In the original patient group there were 512 females and 153 males, but female to male breakdown in the final study group of 353 was not provided*.

*ASD, atrial septal defect; ASO, atrial septal occluder; FUP, follow-up; MH, migraine headache; mos, months; Antithrombotics: ASA, aspirin; AMITRIP, amitriptyline; CLOP, clopidogrel; FNZ, flunarizine; LTG, lamotrigina; PPC, phenprocoumon; PPL, propranolol; IBP, Ibuprofen; PIZ, Pizotofen; TPM, Topiramate; Ticlid, ticlopidine*.

### Study Highlights

#### Preexisting Migraine Change after Percutaneous ASD Closure: Case Studies

Adult patients were involved in all three of the case studies reporting change in preexisting MH (62, 70, 82). Outcomes for all case study patients with preexisting MH were as follows: 3/5 patients (60%) across these case studies experienced change in preexisting MH within hours to 7 days after pASDC, and the remaining two patients experienced change in preexisting MH at 3 months post pASDC. After pASDC, transthoracic echocardiography (TTE) (or TEE) was normal (no evidence of embolization or thrombus) across patients but one patient’s MRI indicated periventricular lesions (62). Abnormal platelet activity was not reported for any patients, and all ASD shunts were resolved within follow-up periods. In three of the case study patients, the change in preexisting MH took the form of heightened MA attack frequency: from two to three times monthly to daily (82); from twice yearly to daily (62), and from once yearly to one to four times monthly [case 3 (70)]. In four case study patients, pASDC attacks decreased in frequency (62) or remitted at final follow-up (70, 82); in one of the patients in which MO was transformed to MA after pASDC [case 1 (70)], MA episodes were reported continuing with a frequency of four episodes monthly at final follow-up (~33 months). Antiplatelet agents modulated MH change after pASDC (here, either increase in frequency or transformation of MO to MA) in a challenge–dechallege–rechallenge (CDR) pattern in two of case study patients [cases 1 and 3 (70)].

#### Preexisting Migraine Change after Percutaneous ASD Closure: Prospective Studies

Luermans et al. (71) reported a reduction in MH prevalence in 23/68 patients (33.8%) with prior MH to a prevalence of 7/57 (12.3%) 12 months after closure, which was a significant reduction (*p* = 0.003). Change in preexisting MH occurred in 15 patients: MH resolution occurred in 8/20 (40%) at 6 months and in an additional 5/18 (27.8) at 12 months after pASDC patients; MH changed from MA to MO in two patients (there were also four incidents of *de novo* MH: see the prospective section for *de novo* migraine prevalence after ASD closure). Riederer et al. (84) reported that of 22 pASDC patients, change in preexisting MH occurred in 7/22 (31.8%) within 6 months from pASDC: in three patients auras disappeared, attack frequency initially increased then decreased to or below baseline in three patients, and persistent MH was resolved in one patient (though twice monthly tension headaches persisted). Overall, there was a reduction in MH frequency from a median of 1.5 before closure to 0.33 attacks per month after closure.

#### Preexisting Migraine Change after Percutaneous ASD Closure: Retrospective Studies

Wilmshurst et al. (19) reported change in preexisting MH in 21/71 patients (30%) after closure. A group taking ASA and clopidogrel, a group that included eight change in preexisting MH patients (and three *de novo* MH patients), had significantly fewer MA attacks (or just incidents of aura) compared to those taking just ASA (*p* < 0.001). Episodes of MH were noted as particularly more frequent and severe in three patients taking ASA alone. Inhibition of migraine and aura events by addition of clopidogrel led the authors to theorize a role for platelet aggregation in the pathogenesis of migraine. Kedhi and Vermeerch (60) reported a drastic level of reduction in MH frequency and severity among 14 patients who underwent pASDC. A Starflex™ occluder was used and it has much lower nickel (nitinol) composition proportion than the Amplatzer™ device. The authors speculated improved rather than exacerbated MH status could have stemmed from absence of a device-triggered nickel alloy allergic response [In a similar vein, Wertman et al. (64) reported evidence of nickel alloy allergic response associated with MH status change after pASDC.] Mortelmans et al. (61) reported change in preexisting MH in 22/75 patients (29.3%): there was an increase in MA prevalence from 8/75 (11%) before pASDC to 11/75 (15%) after closure, but a decrease in MO from 14/75 (19%) before closure to 9/75 (12%) after; MH resolved in 12 patients at least 6 months after pASDC. With respect to the 10 patients in whom MH persisted, frequency was significantly reduced (*p* = 0.01). In addition, while all patients were adults, MH change occurred in preexisting MH patients who were significantly younger than pASDC patients not experiencing MH (*p* = 0.001), and MH change occurred in preexisting MH patients who had larger occluder devices than pASDC patients not experiencing MH (*p* = 0.004). Voet et al. (81), in a follow-up (mean 52 ± 13 months) of the Mortelmans et al. study, reported MH cessation in an additional six patients (*p* = 0.031), meaning an approximate reduction in collective persistent MH patient count from 23/75 (30.7%) before closure (Voet el al reported an additional patient with prior MH) to a post closure late follow-up rate of 16/71 (22.5%). Azarbal et al. (59) reported 7/23 (30.4%) patients with a history of MH; after pASDC, change in preexisting MH occurred in 4/23 (17.4%) patients. Resolution of MH occurred in 3/7 patients (42.9%) (all MA patients) and improved in the single MO patient. The overall changes (which merged ASD and patent foramen ovale patient outcomes) indicated significant levels of MH frequency reduction (*p* = 0.004) and severity reduction (*p* < 0.001). The MIDAS scale was used (IHS criteria were not referenced), and MH changes were noted within a 12-month mean follow-up period. Sharifi et al. (63) reported change in preexisting MH in 5/13 patients (38%) within 40 h post pASDC; MH was characterized as substantially more severe relative to baseline attacks. The attacks (not responsive to ASA, ibuprofen or sumatriptan) were resolved in 4/5 patients (80%) and much improved in the remaining patient within minutes (15 ± 8) of administration of clopidogrel. At 9-month follow-up there was no further occurrence of migraine. Wertman et al. (64) reported 5/42 patients (12%) experienced either change in preexisting MH (characterized as exacerbation) or *de novo* MH after closure. A nickel allergy test, completed in 6 of 42 patients, revealed a positive result in 4/6 (67%), and there was a significant association (*p* = 0.035) between migraine status change (*de novo* MH or change from MH baseline) and positive nickel allergy response. The authors theorized that an inflammatory response from an allergic reaction to nickel (a constituent of the Amplatzer™ occluder) could induce heightened platelet aggregation and embolism with consequent microinfarcts triggering MH. Migraine was reduced or resolved by clopidogrel, and MH prevalence paralleled administration and withdrawal of clopidogrel in a CDR pattern. Migraine was not assessed using IHS criteria, but the MIDAS questionnaire was adopted. The Knepp et al. (52) long-term study (median follow-up 73 months) investigating the safety and efficacy of pASDC using Amplatzer™ occluder in pediatric and adult patients reported cessation of preexisting MH in 2 of two patients within the follow-up period. Kato et al. (76) reported change in preexisting MH after pASDC in 18/20 patients (79%). Within 3 months post procedure, and relative to prior migraine, MH was exacerbated in 11 but improved in 7 patients, which included 1 patient whose MO transformed to MA. Migraine subsequently disappeared in 5/7 patients in the improved group within 3 months from pASDC. Within 12 months from pASDC, 7/11 (63.6%) of the exacerbated group improved and MH resolved completely in 1 patient.

#### *De Novo* Migraine Prevalence after Percutaneous ASD Closure: Case Studies

Adult patients were involved in three of the five case studies reporting *de novo* MH after pASDC (57, 73, 75), and pediatric patients were involved in the remaining two studies (78, 92). All *de novo* case study MH events occurred within 7 days of closure and were type MA. Imaging results within follow-up after closure, including TTE or TEE, were normal (no evidence of embolization or thrombus). One patient’s MRI indicated minor periventricular hyperintensities (73). None of the studies Indicated abnormal platelet activity and the ASD shunts were resolved within follow-up. In four of the case studies (57, 73, 78, 92) antithrombotics ameliorated, reduced, or abolished MA events; in one study, the thienopyridine appeared ineffectual (75). In three studies, MH disappeared at 4 months (92), 6 months (57), and at 9 months (75). However, although MH disappeared in one of the patients, aura without headache persisted at 2-year follow-up (75). In the other two studies MH events continued at 7-year (73) and 2-year (78) follow-up times. Modulation of MH symptom disappearance and re-appearance was most pronounced in one study in particular; there was exquisite response to thienopyridine administration (ticlopidine) and withdrawal in a CDR pattern (73).

#### *De Novo* Migraine Prevalence after Percutaneous ASD Closure: Prospective Studies

*De novo* MH following pASDC was reported in two of three prospective studies (71, 72). Luermans et al. (71) reported *de novo* MH in 4/63 patients (6%) after the procedure. In the overall study result, which included outcome for 23 patients with preexisting MH (see Preexisting MH Change after Percutaneous ASD Closure: Prospective Studies), there was a significant reduction in MH at 12-month follow-up (*p* = 0.003). Wei et al. (72), reported *de novo* MH after closure in 24/40 patients (60%); 18 patients had the first MH within 30 days and the other 6 within 12 months after the procedure (median: 30 days). Consistent with greater occluder size reported in *de novo* MH patients by Mortelmans et al. (61), Wei et al. reported that those experiencing *de novo* MH had larger ASD size (*p* = 0.01) and a greater ASD to occluder length ratio (*p* = 0.03). Of 17 patients whose plasma CGRP was tested before and after closure, 4/17 (24%) experiencing *de novo* MH had lower pre-closure compared to post closure CGRP levels (*p* = 0.042). There was also indication that the CGRP levels increased during MH attack (*p* = 0.03). Authors postulated that larger ASD size and lower pre-closure CGRP could be predictors of *de novo* MH after pASDC. MH remitted spontaneously in 7/24 (29%) of *de novo* MH patients within 6 months of pASDC, but persisted in 17/24 (71%). It was noted that one patient’s MH attacks responded most favorably when ASA was replaced by clopidogrel.

#### *De Novo* Migraine Prevalence after ASD Closure: Retrospective Studies

*De novo* MH was reported in 10 of 13 retrospective studies (19, 46, 51, 52, 58, 61, 64, 67, 76, 93). Yew and Wilson (58) reported *de novo* MH in 2/25 (8%) patients after closure; symptoms were strongly suggestive of MA (IHS MH criteria were not referenced) and interpreted in this review as MA. Pediatric and adult patients were included in the study, and *de novo* MH (categorized as MA in this review) occurred within 1 week after closure in one of the *de novo* MH patients. At final follow-up (60 months) MH episodes continued to occur in one patient but were resolved in the other patient. Wilmshurst et al. (19) reported *de novo* MH in 9/71 adult patients (13%) after closure. As mentioned in the change in preexisting MH section, the group taking ASA and clopidogrel (a group that included three *de novo* MH and eight patients with preexisting MH change) had fewer MA attacks (or just incidents of aura) compared to those taking just ASA (*p* < 0.001). Episodes of MH were noted as particularly more frequent and severe in two *de novo* patients taking ASA alone. As also noted in the section for retrospective preexisting MH change, the inhibition of migraine and aura events by addition of clopidogrel led the authors to postulate a role for platelet aggregation in the pathogenesis of migraine. Mortelmans et al. (61), at 2 months post pASDC, reported *de novo* MH in 9/75 patients (12%); MA occurred in seven and M0 in two patients. At 6 months after pASDC, *de novo* MH was reported in 10/75 patients (13%); MA in seven; and MO in three patients. *De novo* MH patients were significantly younger (29 ± 18 vs. 58 ± 17) than pASDC patients not experiencing MH (*p* = 0.045), and *de novo* MH patients had larger occluder devices than pASDC patients not experiencing MH (*p* = 0.01). Voet et al. (81), in a follow-up (mean 52 ± 13 months) of the Mortelmans et al. study, reported MH cessation in six *de novo* MH patients (*p* = 0.03); this study also reported cessation in six patients with preexisting MH. As previously noted (see Preexisting Migraine Change Retrospective Studies) Wertman et al. (64) reported 5/42 patients (12%) experienced either change in preexisting MH (characterized as exacerbation) or *de novo* MH after closure (*de novo* vs. prior MH outcomes were not distinguished, and were reported in Table 2 conservatively as two patients with change in preexisting MH and two with *de novo* MH). Additional highlights of this study are outlined in the preexisting MH section reviewing retrospective studies. Providencia et al. (94) reported *de novo* MH in 3/25 patients (12%), each of whom had a morphological trait (short aortic rim) that significantly differed from other patients not experiencing MH after closure (*p* = 0.036). Migraine resolved in 2/3 patients (67%) within 12 months. Rodés-Cabau et al. (46) reported *de novo* MH in 13/112 patients (12%) after pASDC. Inception of *de novo* MH occurred in 6/13 patients (46%) within 7 days of pASDC; MA occurred in five of these patients and MO in one patient. While MH spontaneously disappeared in 4/13 *de novo* MH patients (31%), it continued in 9/13 (69%) at 2 years post pASDC, though frequency of MH had significantly reduced relative to frequency at the 3-month follow-up (*p* = 0.032). The author’s speculated that, for some patients, pASDC may act as permanent MH trigger. Pediatric and adult patients were included (age range 16–60; mean 39 ± 19). Patients developing MH were significantly younger (*p* = 0.02), tended to be female, and pASDC was the sole independent predictor of MH (odds ratio 7.7; *p* = 0.01). The emphasis in three studies (51, 52, 93) was assessment of pASDC safety and efficacy; MH status was also reported. Li et al. (93) investigated the safety and efficacy of TTE (as opposed to TEE) guided pASDC. Reporting a 96.9% rate of successful TEE-guided closure, *de novo* MH occurred in 3/188 patients (2%) after closure, 2/3 (67%) of which occurred within 7 days of pASDC; IHS criteria were not referenced. Pediatric and adult patients were included, and pASDC patients were divided in to three groups based on ASD diameters (5–14 mm, *n* = 66; 15–20 mm, *n* = 60; and 21–38 mm, *n* = 65). Two of the *de novo* MH patients where from the group with the largest ASD diameter, the other *de novo* MH patient was from the group with an ASD diameter in the 15–20 mm range. Migraine events resolved in all three patients within follow-up. Complications were reported within the group with the largest occluder devices (these patients were not specified as experiencing *de novo* MH): devices in three patients migrated and required cardiac surgery; one patient developed a device thrombus that resolved over 10 days of intensified anticoagulant administration. Knepp et al. (52), in a long-term study (median follow-up 73 months; 60% response rate) investigating the safety and efficacy of pASDC using Amplatzer occluder in pediatric and adult patients, reported *de novo* MH in 4/94 of patients (4%) after pASDC. Resolution of the four cases of *de novo* MH was not specified; non-resolution was assumed (persistent MH cessation was also reported in two patients with a prior history of MH as noted in the preexisting MH retrospective section). While there was a 97% rate of successful ASD closure, one child died of a cerebral vascular accident at 18 months post closure. Vijarnsorn et al. (51) also reported long-term outcomes (12 months after closure) following pASDC with the Amplatzer occluder. Patients were divided into pediatric, adult, and older adults groups. *De novo* MH occurred in 19/353 of patients (5%) within 12 months of closure; *de novo* MH prevalence did not significantly differ between groups and ranged from 4 to 8.3% across all 3 age groups. There was a 93.4% rate of ASD closure, no mortalities, but greater atrial fibrillation/flutter as well as chest discomfort in older adults at 12 months after closure (*p* < 0.05). A single incident of eye embolism, which resolved within 3 months post procedure, was also reported. In the largest study to date focusing on the potential impact on migraine of pASDC, Kato et al. (76) reported *de novo* MH in 23/207 patients (11%) after closure (13 MA; 10 MO). Pediatric and adult patients were included in the study, and compared to the age of pASDC patients who did not experience post closure MH (M = 27 ± 22), *de novo* MH patients were significantly younger (M = 15 ± 11; *p* = 0.03). Within 7 days from pASDC, 13/23 (57%) *de novo* MH incidents occurred and of these 10 were MA and 3 MO. Administration of thienopyridines (ticlopidine or clopidogrel) improved (reduced frequency) MH in 6/23 patients (26%). At final follow-up (45 ± 23 months), *de novo* MH resolved in 8/23 patients (35%), improved in 14/23 patients (61%), but nevertheless continued at final follow-up in 15/23 patients (65%); a pattern consistent with other research (46, 58, 61, 73, 78). Speculating in parallel with other research (46), it was suggested that occluder implantation could act as a permanent MH trigger. Post closure replacement of ASA with either ticlopidine or clopidogrel reduced MH frequency and aborted continuous MH events.

#### *De Novo* Migraine Prevalence after ASD Closure: Randomized Controlled Trial

In the first randomized controlled trial Rodes-Cabau et al. (79) investigated the efficacy of clopidogrel to prevent *de novo* migraine after pASDC. Patients (*n* = 171) with no history of migraine were randomized to one of two groups: ASA plus a placebo (*n* = 87; the control group) or ASA plus clopidogrel (*n* = 84; the clopidogrel group). *De novo* MH occurred in 27/171 patients (16%) within 3 months following the procedure (14 MA; 13 MO); *de novo* MH occurred in 16/27 patients (59%) within the first week after pASDC. The ASA plus clopidogrel group had significantly fewer *de novo* MH incidents (*p* = 0.03) and a significantly lower MIDAS scale severity rating (*p* = 0.046). Of 155 patients adherent to antithrombotic treatment (control group *n* = 79; clopidogrel group *n* = 76), incidence of MH attack within 3 months was 19/79 in controls (24.1%) compared to 7/76 in the ASA plus clopidogrel group (9.2%)—an approximately 15% significant (*p* = 0.02) reduction of migraine incidents. In addition, within the 3 months after pASDC the clopidogrel group had less than half the number of MH days compared to the control group (*p* = 0.02).

## Discussion

This review encompassed 25 studies and 1,646 patients who underwent pASDC between May 2003 and November 2015 and investigated the number of patients experiencing MH status change subsequent to pASDC. Because of a difference in prevalence, publishing bias, or both, *de novo* MH data were reported in more studies (18/25) more comprehensively than change in preexisting MH (12/25 studies). For example and as noted in Section “Results,” a breakdown of change after implantation in preexisting MH differentiated by either by gender or MH type was not available, though additional categorical breakdown by gender and type was available for 52 and 80% of *de novo* MH patients, respectively.

As a prelude to discussion of the results and as noted in the introduction, this review focuses on MH status change, change in preexiting MH or the rise of *de novo* MH, within the context of a reparative coronary procedure, pASDC. Because of this context, caution should be exercised in generalizing findings and the implications thereof to migraine pathophysiology in the general population.

After pASDC and by final follow-up, MH changed (most often in frequency or severity) in 72% of patients (91/126) with a history of preexisting MH, and more strikingly debuted in between 10 (153/1,520) and 18% (153/836) of patients with no prior history of MH. The overarching finding was the modulation of migraine status evident in a substantial proportion of patients (see Table 1 for a summary) within hours to months after implantation. The increase and decrease of migraine incidence appeared in step with initial heightened proinflammatory but months later reduced inflammatory stages of endothelialization. The most apparent modulators were the procedure itself and antithrombotic administration, each of which was often temporally juxtaposed to MH status change. The temporal link to the procedure was underlined by the relatively high percentage of *de novo* MH occurrence within the 7-day post procedure interval (34%); the temporal link to antiplatelet agents was evident in the rapid modulation of migraine associated with administration in 11/25 studies.

### MH Status Change: Timing and Type of MH

The count of patients experiencing MH status change was obtained for two time points: the interval within 7 days after implantation and at final follow-up, the latter ranged from 3 to 73 months (Mdn = 12). The substantial impact of the procedure itself was underlined by the early post implantation change in MH status. While more than a few studies (63, 65, 69, 70, 75, 77, 78, 80) reported MH change within hours of the procedure, by 7 days after implantation 12% (11/91) of patients with preexisting MH underwent MH change (mainly in frequency or severity), and the initial onset of *de novo* MH occurred in 34% (52/153) of *de novo* cases. While type of MH was not differentiated in 20% of *de novo* MH patients (31/153), data that were available indicated *de novo* MA within 7 days from pASDC accounted for MH debut in 48% of *de novo* MH cases (25/52) and *de novo* MO within 7 days from implantation accounted for MH debut in 12% of patients (6/52). Relative to the 33% prevalence of MA in the general population (95), by final follow-up the rate of total *de novo* MA patients after pASDC was high, at 58% (71/122); the rate of MO was 42% (51/122). Even in the unlikely event that undifferentiated MH in all *de novo* patients fell in the MO category there would be 54% MO (82/153) and 46% MA (71/153) and hence still a disproportionately large percentage of MA relative to the general population prevalence. By final follow-up, change in preexisting MH occurred in 72% of patients (91/126), and *de novo* MH occurred in 10–18% of patients with no history of MH. Also within follow-up, there was improvement in 14% of patients with preexisting MH and resolution in 37% (47/126) of those with preexisting MH. With respect to *de novo* MH, MH in 10% of patients (16/153) improved and in 24% (37/153) MH resolved. Improvement or resolution occurred in 52% of patients (65/126) with a prior history of MH within follow-up, but nevertheless in 63% of patients (79/126) with a prior history of MH, episodes continued beyond follow-up. Improvement or resolution occurred in 35% *de novo* MH patients (53/153) within follow-up, but in 76% who experienced *de novo* MH (116/153) migraine continued beyond follow-up.

### MH Status Change and Antiplatelet Agents

Effective modulation of MH status by antiplatelet agents, was expressly reported in 11/25 or 44% of studies (see Results). ASA was used in 21/25 (84%) studies, and the thienopyridine clopidogrel was used in conjunction with ASA in 12/25 (48%) of studies. Two studies, including a RCT, indicated significantly reduced MH frequency in those taking dual antiplatelet therapy (ASA plus clopidogrel) relative to ASA alone (19, 79). In addition, some data indicated an exquisite MH response in CDR fashion to antiplatelet agents (64, 70, 73).

### Relevance of Gender, Defect Size, and Age

As reported in Section “Results,” patient counts of change after pASDC in preexisting MH differentiated by gender (as well as MH type) was not available from the literature, and *de novo* MH incidence differentiated by gender was available for just 52% of new-onset cases. The available data indicated 80% of *de novo* MH occurred in females and 20% in males, with MH debut by gender not specified in 48% of *de novo* MH patients, though it is likely most *de novo* MH cases not differentiated by gender were female patients given the disproportionately high female to male ratio of pASDC patients. Why ASD are more common in females than males (53) is unknown, but higher incidence of MH in females (see the introduction) than males may be related to sex-linked differences in platelet reactivity. Platelets have demonstrated greater reactivity in women than men in response to adenosine diphosphate (96, 97), which is a known proinflammatory substance that would normally be released in higher concentrations during the pASDC procedure and during the subsequent period of endothelialization. Platelet reactivity is likely influenced by hormones (particularly estrogen fluctuation in women associated with menstruation, the postpartum period, hormone replacement therapy, and menopause), but the actual role of hormones in platelet biology remains undecided (98).

Three studies reported anatomical traits, which may have predisposed patients to *de novo* MH or exacerbated preexisting MH. One study reported short aortic rim in *de novo* MH patients (68); another study reported significantly greater ASD/occluder size in both patients with preexisting MH change and in *de novo* MH patients (61); an additional study reported significantly larger ASD size and ASD length to occluder length ratio in *de novo* MH patients (72). Otherwise, clinical characteristics (e.g., Qp/Qs, ASD size, occluder size, etc.) did not differ significantly between those who did and those who did not experience pASDC migraine status change.

Occurrence of *de novo* MH has been reported to not differ significantly between pediatric and adult patients (51), though, contrarily, other research reports higher *de novo* MH prevalence among pediatric patients (46, 77) and younger adult patients (46, 61). Some data indicated adults who underwent change in preexisting MH were younger that those in whom MH did not persist or develop (61).

### Potential Mechanisms

Differing theories have been advanced regarding the nature of pathophysiological change and migraine following pASDC and posited mechanisms can be differentiated into microembolic, biochemical, and allergic response categories, though migraine status change in the context of pASDC likely involves interactions among such potential agents.

### Microembolism

The known association between cardiovascular risk factors (e.g., pre-clinical brain lesions and ischemic stroke) and MA is well documented [Elliott (99); Kruit et al. (36); Kurth et al. (38); Kruit et al. (35); Schurks et al. (39); Bigal et al. (34); Kruit et al. (37)]. Several other vascular risk factors have been reported as associated with migraine: experimental hypoxia (100); altered brachial and femoral artery compliance as well as decreased brachial artery diameter (41); increased carotid artery media thickness, a subclinical marker of early atherosclerosis (43, 44), has been reported as heightened in pediatric migraineurs (45) and adult migraineurs (42). Migraineurs (particularly women) with MA have two times the risk of ischemic stroke (38), and MA and migraine in general, in an unadjusted population-based analysis, have been associated with stroke, myocardial infarction and claudication (34). The implication is that inflammatory response, related to vascular conditions, is a potential trigger of SD, at least as concerns MA.

The current review data showed a disproportionately high percentage of MA among patients experiencing *de novo* MH but evidence of device thrombus was rare, just two incidents in 1,646 patients (52, 93). In research not focusing on MH, the largest study assessing device thrombus formation after ASD percutaneous closure (101) found thrombus formation occurred in 1.2% (5/407) of ASD patients, which is a low rate but nevertheless about 10 times higher than that in the current review. However, the latter study assessed thrombus formation for six occluder device types including the Amplatzer™ device; the Amplatzer™ device actually had a 0% incident rate of device thrombus formation and the other device types accounted for the 1.2% incidence of device thrombus formation. The Amplatzer™ device was used in more than 95% of patients in the current review so the 0% rate of device thrombus evidence with respect to the Amplatzer™ occluder reported by Krumsdorf et al. is quite consistent with the very low incidence of device thrombus reported in the present review. Moreover, in the current review, the presence of lesion was evident in only one patient (62); and there was no evidence of abnormal platelet activity reported—observations that do not favor an ischemic-related trigger theory of MA.

However, as noted in the introduction, research in mice has demonstrated subclinical microembolism triggers SD, the substrate of MA. Air bubble or particles (micro-spheres) were introduced into the carotid circulation; but these microemboli did not result in tissue infarction, did not leave histological evidence of tissue damage or inflammation response (17). Microembolic theories of migraine are not new but do appear to be gaining growing favor (102, 103). Moreover, in another type of ASD known as a patient foramen ovale (PFO), venous matter unfiltered by the lungs can access left side of the heart (and hence the brain) via an aberrant conduit that forms a right-to-left interatrial shunt. An ASD has the opposite left-to-right shunt property except during instances where right atrial pressure exceeds the left [such as during a sneeze or Valsalva maneuver]. PFO is unambiguously linked to cryptogenic stroke (104), injected microbubbles can trigger MA in patients with symptomatic PFO (105, 106), and MA has been triggered with high consistency by injection of sclerosing agents, which induce an inflammation response, in those with a PFO (107, 108). Just as the RLS mechanism of PFO can introduce potential subclinical microembolic and inflammatory substances into the left side of the heart, the pASDC procedure and subsequent endothelialization induces potential subclinical microembolic and proinflammatory elements on the left side of the occluder that have access to the brain.

### Allergic Response

The amount of nickel titanium (nitinol) in a given brand of occluder device may vary but it is nevertheless a common denominator in most occluder devices (109). While reported incidence of nickel allergic response related to implanted occluders is rare, a 5-year study involving 131 PFO patients found post implantation chest discomfort (and palpitations) was significantly greater in those with nickel hypersensitivity (110). In a patient with confirmed general metal hypersensitivity, extreme exanthema 3 days after percutaneous PFO closure necessitated explantation of the implanted device, and 3 days after device removal skin lesions resolved (111). A case of severe contact dermatitis erupting the day after percutaneous closure of a patent ductus arteriosus has been described in a patient with a confirmed nickel allergic response; several days subsequently, the dermatitis disappeared after the administration of prednisone (112). Only one study in the current review reported evidence of a significant association between migraine status change and a positive nickel allergy response in 67% (4/6) patients (64).

### Biochemical Constituents and Platelets

The apparent absence of abnormal platelet dynamics in the data for this review likely reflects the efficacy of antiplatelet agents. Some research involving PFO (RLS) and MH has shown reduction or resolution of MH, mostly MA, using clopidogrel (113), but a randomized control pilot project reported that clopidogrel had no favorable effect on MH (114). The sole pASDC RCT in the current review reported the addition of clopidogrel was associated with a greater than 50% reduction in the burden of MH days as well as a significant reduction in MH incidents (79).

As already noted, 11/25 studies in this review expressly reported the favorable response of MH to antiplatelet agents. Platelets contribute to the generation of microembolism (115, 116) and heightened aggregation of platelets has been demonstrated in migraineurs (117) and after pASDC (118). Aggregating platelets normally release a number a substances including thromboxane A2, adenosine diphosphate and serotonin. In addition, serotonin released from aggregating platelets serves as a vasoconstrictor, but under normal conditions, and as aggregation subsides, reduced levels of serotonin can lead to vasodilation (119). It has been hypothesized that alteration in plasma serotonin level causes MH (MO or MA), and that platelet aggregation and heightened serotonin induce vasoconstriction causing reduction in blood flow and the neuronal manifestation of aura (14). Other research, already cited, argues that pro-inflammatory factors (including platelet aggregation and serotonin) may trigger the neuronal manifestation of MA (17, 102), which is SD. It follows that subclinical microembolism, derived from a proinflammatory cascade including platelet aggregation, plays a role in MH status change after pASDC. Also associated with migraine episodes are up-regulated inflammatory platelet and white blood cell (leukocyte) aggregates as well as cytokines (e.g., interleukins 1, 6, and 8 and tumor necrosis factor-α); altered nitric oxide concentrations have also been found during MH attacks (15). In addition, and as mentioned in the introduction, elevated plasma endothelin (ET-1), a marker for endothelial injury, was recently reported in those with migraine (42).

Sumatriptan, the serotonin (5-TH) receptor agonist, has been presumed to relieve MH pain by interfering with 5-HT receptors on trigeminal nerve endings (120). The neuropeptide and potent vasodilator CGRP is also influenced by administration of sumatriptan: CGRP levels are reduced and migraine pain can be effectively subdued (121). As noted in the introduction, antithetically, migraine pain can be induced by intravenous introduction of CGRP (30). The capacity of CGRP’s level to alter migraine pain argues for its role as a mechanism in the trigeminovascular nociceptive pathway. One study within the current review reported 24% of patients (4/17) with *de novo* MH had significantly lower pre-closure relative to post-closure CGRP levels, Wei et al. (72). Treatment with sumatriptan was not reported. Moreover, post closure CGRP and treatment with triptans was scarcely reported across all studies. In preexisting MH patients CGRP levels were not reported and triptans were administered to few patients (7/1,646). With respect to *de novo* MH patients, and with the exception of the Wei et al. study, CGRP measurement was not reported and administration of triptans was reported for only a few patients (3/1,646). Considering that this review assessed incidence of migraine status change within the bounds of pASDC, and the primary procedure-related adverse outcome concern is thromboembolism, it should be expected that antithrombotic and antiplatelet agents constitute the main prophylaxis measure. However, given the potential inhibitory effect of CGRP antagonists on SD (22), and this neuropeptide’s apparent integral role in migraine pain management, recent advances in CGRP antagonist research and development (33) auger well for the future contribution of CGRP antagonists in the treatment of migraine, both in general population and potentially for management of migraine pain within the context of migraine after pASDC.

### Endothelialization: A 3-Month Process?

The overall pattern of MH early intensification followed by later amelioration seems to roughly parallel stages of early implantation and progressing endothelialization. The initial post procedure intensification of MH might be the expected outcome of the inflammation response to the trauma of occluder implantation with heightened early proliferation of platelets and other proinflammatory potential thromboembolic elements. The 34% rate of patients with *de novo* MH constrained within hours to 7 days from pASDC seems to exemplify early post procedure intensification of MH status change. After days, weeks and months of device endothelialization, reduced inflammation response would be expected as the healing process progresses. The estimated time to complete endothelialization as derived from animal (swine) research is 3 months (122), and it could be assumed that proinflammatory substances and microembolism related to endothelialization are therefore an unlikely cause of MH after this length of time. However, it has been demonstrated that complications from late, incomplete endothelialization can occur including coronary and cerebral embolism at 5 and 8 years after device implantation (123, 124). Accordingly, proinflammatory derivatives of incomplete endothelialization may be the trigger of MH events persisting beyond the previously estimated 3-month completion time of endothelialization. In the current review, this may underlie the high percentages of patients with MH persisting after final follow-up: 63% of preexisting MH cases and 76% of *de novo* MH cases. In five patients, MH persisted 2 years (78), 5 years (58), and 7 years (73) after pASDC.

Evidence of complications associated with incomplete endothelialization are rare but have been reported (124, 125). In a study of patients requiring explantation of percutaneous implants all showed so called neo-endothelialization, and 56% of the patients (5/9) had recurrent thromboembolic events prior to explantation; 2 of the 5 had recurrent coronary and cerebral embolism prior to explantation (124). An improvement in device technology that would minimize the need for explantation, at least where proinflammatory reaction stems from metal allergic response, would be the development of biodegradable devices, though such devices have not yet been formally approved (109).

The development of *de novo* MH is obviously a negative and undesirable side effect associated with pASDC, which underlines the need for continued scrutiny of procedure associated variables. The potential occurrence of *de novo* MH in this context, which ranged between 10 and 18% in the current review, should be weighed against overall prognosis of MH after pASDC and of course the success rate of closure. In 19/25 or 76% of studies (and in most patients) resolution, improvement or indications for momentum in the direction of MH improvement was indicated within follow-up and the frequency and severity of both *de novo* and preexisting MH tended to diminish gradually approaching final follow-up from an early post pASDC peak (46, 52, 57–59, 61–65, 68, 70–72, 75, 77, 82, 85, 93). In addition, high rates of successful ASD closure (a 93% rate estimated across the data) and very low complication rates but rapid recovery have become earmarks distinguishing pASDC. Considering just known complication rates (combined major and minor), the surgical method of ASD closure has a complication rate of approximately 44% compared to 6.9% for the percutaneous method (126).

Yet, the high rate of persistence of MH beyond follow-up, particularly conspicuous in *de novo* occurrences, is an obvious drawback of pASDC. While isolating the cause of *de novo* MH in the context of post pASDC and accounting for MH perpetuation after a median 12 months (range 3–73 months) follow-up is beyond the scope of this review, a proinflammatory trigger is suggested by three main findings. First, research has demonstrated an association of MA with ischemic stroke and with proinflammatory elements including subclinical microembolism (see subsections *Microembolism* and *Biochemical constituents and platelets*). In the data reviewed, MA emerged as the dominant type of *de novo* MH. Second, and as already noted, research has found higher aggregation of platelets in migraineurs (117) and after pASDC (118). The relatively high efficacy of antiplatelet agents expressly reported in 11/25 studies reviewed argues for a role of platelets (platelet aggregation, platelet-leukocyte aggregation, platelet serotonin secretion, etc.) in migraine and for the validity of antiplatelet agents as a migraine prophylaxis. Third, and perhaps most significantly was the timing of MH status change: the wax and wane of MH loosely paralleled early post procedure intensified (heart tissue) inflammatory response and the subsequently diminished inflammatory response occurring at final follow-up, the latter ostensibly associated with advanced endothelialization.

While further studies are needed to determine the optimal duration for antiplatelet administration, the persistence of *de novo* MH in a large proportion of patients beyond final follow-up evident in the data suggests an extended period of antiplatelet agent use following pASDC is warranted. In addition, because antiplatelet agents are generally not without some negative side effects (such as increased bleeding), for non-acute, longer-term application consideration should be given to use of alternative non-standard antiplatelet agents (e.g., nattokinase) that may have lesser side effects yet potentially prove sufficiently effective. While assessment of efficacy of such alternative stream antithrombotic agents is outside the extent of this review, one indicator will be the outcome of a clinical trial currently underway for nattokinase (https://clinicaltrials.gov/ct2/show/NCT02080520).

### Limitations

The main limitation of this review was reliance on frequency qualitative data, which is a restrictive and unsophisticated measure. While some quantitative data was available, such data was either too scarce or inappropriate to measure patient prevalence of change in MH status after pASDC. Other limitations include a possible MH incident recall bias given 13/25 studies were retrospective, some inconsistency in use of IHS (56, 127) diagnostic criteria (17/25 studies used IHS criteria), and the virtual absence of testing for resistance to the most used theophylline clopidogrel. A positive test for resistance could result in an under estimate of this antiplatelet agent effectiveness. It also warrants mention that inconsistency in use of IHS criteria by medical or research personnel without neurological expertise could have resulted in misdiagnoses of migraine, the extent of which can not be determined from the data.

## Conclusion

The studies reviewed revealed change in MH—a neurological event—following a vascular corrective procedure. Between 10 and 18% of patients experienced *de novo* MH and 72% of patients with preexisting MH experienced a change in MH status after pASDC. In 19/25 studies intensification of change in MH status in the initial, hours, weeks and months after pASDC was followed by amelioration of MH as the post operative timeframe approached 6–12 months; a pattern that seems to loosely parallel stages of endothelialization: early heightened proinflammatory response and the reduction of the latter in later near-completion stages of endothelialization. This lends further support to the contention that proinflammatory response factors (including, aggregated platelets, serotonin, ADP and microembolism) trigger MH status change after pASDC. This contention is additionally reinforced by effective MH treatment by antiplatelet agents expressly reported in 11/25 studies, and by the emergence of MA as the dominant *de novo* MH type, its known association with ischemic stroke also implicating proinflammatory possibly microembolic, undetected, triggers of MH. Despite general indications for gradual MH amelioration, there were high rates of MH persistence, particularly *de novo* MH, beyond the 12-month median follow-up time. Other research cited has demonstrated incomplete endothelialization extending to several years after pASDC. Progressing but on-going endothelialization could explain MH amelioration at final-follow-up but nevertheless continued persistence beyond final follow-up.

## Author Contributions

Both authors (CL and JD) shared in the acquisition and interpretation of data as well as in the revising and approval of this work for publishing.

## Conflict of Interest Statement

This research did not receive funding from any specific agency (non-profit or commercial). The authors declare there were no financial or other relevant interests that relate to the research described in this paper.

## Abbreviations

ASD, atrial septal defect; IHS, International Headache Society; MH, migraine headache; MA, migraine with aura; MO, migraine without aura pASDC, percutaneous atrial septal defect closure; PFO, patent foramen ovale.
