# Supplementary material for: Migraine Modulation and Debut after Percutaneous Atrial Septal Defect Closure: A Review
Source: Front Neurol. 2017 Mar 20;8:68. doi: 10.3389/fneur.2017.00068 (PMC5357661; doi:10.3389/fneur.2017.00068)
Supplement: Supplementary file 1 [file Table_1.DOCX]

Supporting Information

Table S1.

| Study *n* | PEMH  Hx | PEMH CHG | PEMH CHG  Wi 7d from pASDC | PEMH Impr Wi FUP | Prior  MH Res Wi FUP | De novo MH | De novo MH F | De novo MH M | De novo  MA | De novo MO | De novo MH Wi 7d from pASDC | De novo MO Wi 7d from pASDC | De novo MA Wi 7d from pASDC | De novo  MH Impr Wi FUP | De novo MH Res Wi FUP |
| --- | --- | --- | --- | --- | --- | --- | --- | --- | --- | --- | --- | --- | --- | --- | --- |
| PC F  1 5  3 2  13 1  14 1  22 1  23 1  25 2  40 1  42 1  63 1  71 1  75 1  94 1  97 1  112 1  171 1  188 1  207 1  353 1 | PC F  0 10  1 2  2 2  3 1  5 1  7 1  10 1  21 1  22 1  23 1  29 1  NA 3 | PC F  0 9  1 2  2 2  3 1  4 1  5 1  7 1  12 1  15 1  18 1  21 1  NA 4 | PC F  1 3  2 1  3 1  10 1  NA 19 | PC F  1 3  2 1  3 1  10 1  NA 19 | PC F  0 1  1 1  2 2  3 2  5 1  6 1  12 1  13 1  NA 15 | PC F  0 7  1 3  2 2  3 3  4 3  9 1  10 1  13 1  19 1  23 1  24 1  27 1 | PC F  0 5  1 4  2 3  3 1  11 1  18 1  21 1  NA 9 | PC F  0 9  1 3  2 1  3 2  5 1  NA 9 | PC F  0 6  1 3  2 4  4 1  7 1  9 1  13 2  14 1  NA 6 | PC F  0 10  1 1  2 1  3 1  4 1  7 1  10 1  11 1  13 1  NA 7 | PC F  0 1  1 4  2 1  3 1  4 2  6 1  13 1  16 1  NA 13 | PC F  0 5  1 1  2 1  3 1  NA 17 | PC F  0 1  1 4  2 1  4 1  5 1  10 1  NA16 | PC F  0 2  2 1  14 1  NA 21 | PC F  0 2  1 3  2 1  3 1  4 2  6 1  7 1  8 1  NA 13 |
| 1646 | 126 | 91 | 11 | 18 | 47 | 153 | 63 | 16 | 71 | 51 | 52 | 6 | 25 | 16 | 37 |

Categories are indicated in the first row, frequency distributions in the middle row, and totals are on the bottom row. De novo MH= new onset migraine; F= frequency; Impr= improved; NA = data not available or inapplicable; PC= patient count; PEMH Hx= history of migraine prior to pASDC; pASDC = percutaneous atrial septal defect closure; Res = resolved; Study *n*= number or frequency of studies with *n* patients per study; Wi =within; 7d = 7 days.
